# Non-contact hemodynamic imaging reveals the jugular venous pulse waveform

**DOI:** 10.1038/srep40150

**Published:** 2017-01-09

**Authors:** Robert Amelard, Richard L. Hughson, Danielle K. Greaves, Kaylen J. Pfisterer, Jason Leung, David A. Clausi, Alexander Wong

**Affiliations:** 1 Systems Design Engineering, University of Waterloo, Waterloo, N2L3G1, Canada; 2 Schlegel-University of Waterloo Research Institute for Aging, Waterloo, N2J0E2, Canada; 3 Applied Health Sciences, University of Waterloo, Waterloo, N2L3G1, Canada

## Abstract

Cardiovascular monitoring is important to prevent diseases from progressing. The jugular venous pulse (JVP) waveform offers important clinical information about cardiac health, but is not routinely examined due to its invasive catheterisation procedure. Here, we demonstrate for the first time that the JVP can be consistently observed in a non-contact manner using a photoplethysmographic imaging system. The observed jugular waveform was strongly negatively correlated to the arterial waveform (*r* = −0.73 ± 0.17), consistent with ultrasound findings. Pulsatile venous flow was observed over a spatially cohesive region of the neck. Critical inflection points (*c, x, v, y* waves) of the JVP were observed across all participants. The anatomical locations of the strongest pulsatile venous flow were consistent with major venous pathways identified through ultrasound.

Cardiovascular disease is the leading cause of mortality, resulting in 17.3 million deaths per year globally, and a third of all deaths in the United States^1^. Cardiovascular monitoring is essential to assessing and maintaining or enhancing quality of life through preventive and acute care. The jugular venous pulse (JVP) waveform is a powerful diagnostic tool for assessing cardiac function. The jugular vein is a major venous extension of the heart’s right atrium, so changes in atrial pressure are reflected in the jugular waveform. Distortions in the JVP provide insight into cardiac function without direct assessment of the heart itself, such as resistance diseases (e.g., pulmonary hypertension, tricuspid stenosis^2^,^3^), mechanical diseases (e.g., tricuspid regurgitation^3^), electrical diseases (e.g., atrial fibrillation, heart block, atrioventricular dissociation^3^), abnormal external forces (e.g., tamponade, constrictive pericarditis^3^,^4^), and heart failure^5^.

A primary problem with assessing JVP lies in the current standard method of measurement: invasive catheterisation. Catheterisation requires surgically inserting a central line into the jugular vein, superior vena cava, or right atrium. This is an invasive procedure requiring surgical expertise. Therefore, although the JVP can provide important clinical insights, JVP examination is not routine and is only performed when there is probable cause for monitoring. Additionally, since catheter monitoring is limited to measuring a single location, spatial flow perfusion characteristics cannot be assessed, which may encode important clinical information^6^,^7^. Ultrasound has recently been proposed to measure the JVP through Doppler velocity imaging^8^,^9^. However, these methods require constant stable probe skin contact, expensive ultrasound equipment, trained ultrasound technicians, and is only able to provide axial hemodynamic information.

Photoplethysmographic imaging (PPGI) systems are non-contact biophotonic cardiovascular monitoring systems that may provide touchless, non-invasive JVP monitoring. Building on photoplethysmography theory^10^, PPGI systems comprise a camera and an illumination source, and leverage the properties of light-tissue interaction to assess blood flow in the skin. These systems have recently gained interest due to their non-contact light-based nature. However, use of PPGI systems have been limited to extracting high-level vital signs, such as heart rate^11^,^12^,^13^,^14^,^15^ and respiratory rate^13^,^16^. The few studies that have reported spatiotemporal perfusion have been limited to perfusion through the hand in constrained environments^17^,^18^. Analysing the spatiotemporal trajectory patterns in the neck can provide insight into whether PPGI can be used to detect the JVP. To this end, we know of no publication that has investigated the use of PPGI systems for JVP monitoring. In fact, we know of no study that has explored leveraging PPGI to assess venous blood flow. Given the clinical promise of non-invasive JVP assessment, we are motivated to investigate the feasibility of biophotonic observation of the jugular pulse in the neck, as well as information about its spatial perfusion patterns.

In this exploratory study, we investigated the feasibility of non-contact biophotonic JVP monitoring by assessing the spatiotemporal pulsing patterns in the neck in 24 participants. Strong pulsatile flow was successfully observed, consistent with ground truth finger photoplethysmography measurements. Two different types of pulsatile flow were observed in all participants: one that corroborated with the ground truth arterial pulse, and one that exhibited strong inverted pulsing characteristics. The hypothesis was that the two pulsatile flows were major arterial and venous blood flow waveforms. To test this hypothesis, the spatial properties of the pulsing were compared to the carotid artery and jugular vein track found through ultrasound, and the waveforms were compared to the JVP. Major venous flow was observed in all participants. Here, we demonstrated for the first time, to our knowledge, that the JVP could be assessed with a PPGI system. Furthermore, the jugular pulse waveform was observed at many different locations along the venous track, providing indication of the pulse trajectory. These fundamental findings may be leveraged in the future as a new non-contact monitoring method to assess cardiac function.

## Results

### Detection of pulsatile flow

Table 1 shows the sample summary demographics. The sample consisted of a wide range of ages (9–60 years), body types (BMI 16.4–35.1 kg · m^−2^), and fair distribution of gender (11/13 female/male).

**Figure 1:**
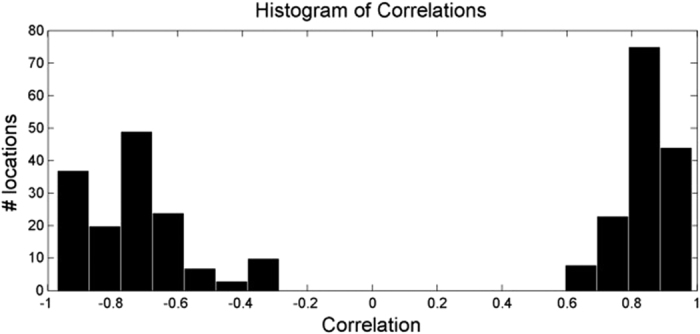
Histogram of the strongest signal correlation values relative to ground truth arterial waveform across all participants (*n* = 24). The distribution is strongly bimodal, indicating one group of signals that are highly correlated with the arterial waveform, and another group that is strongly negatively correlated with the arterial waveform.

To test the base hypothesis that the imaging system was able to consistently detect localised pulsatility in vascularised locations, each 5 × 5 mm region was analysed for pulsatile blood flow. The strongest 2.5 cm^2^ tissue area of positively and negatively correlated pulsatile regions were identified, totalling 5 cm^2^ tissue area. Figure 1 shows the correlation distribution of the strongest 5 cm^2^ total tissue, across all participants. Pulsatile flow was consistently observed across all participants. The distribution is strongly bimodal, indicating both strong arterial (*r* = 0.85 ± 0.08) and inverted (*r* = −0.73 ± 0.17) pulse signals relative to the ground truth PPG. That is, some signals exhibited strong positive correlation to the PPG waveform, whereas other signals exhibited strong negative correlation to the PPG waveform. Strong signals (|*r*| > 0.5) were found in most participants. The weakest signals were found in participants with high body fat content and age-related skin inelasticity.

**Figure 2:**
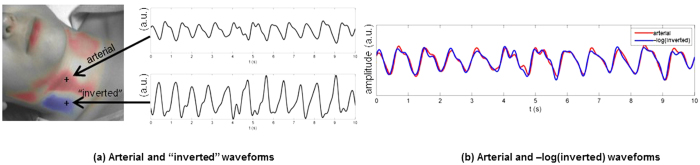
Comparison of scale-normalized arterial and inverted pulse waveforms in a typical example. (**a**) The participant exhibited strong arterial pulsations, characterised by a sharp rise to systole and a dicrotic notch, as well as strong inverted pulsations, characterised by a phase-offset gradual rise and sharp drop. (**b**) The arterial and inverted waveforms are strongly linked through an inverse relationship (*r* = 0.96). Best viewed in colour.

**Figure 3:**
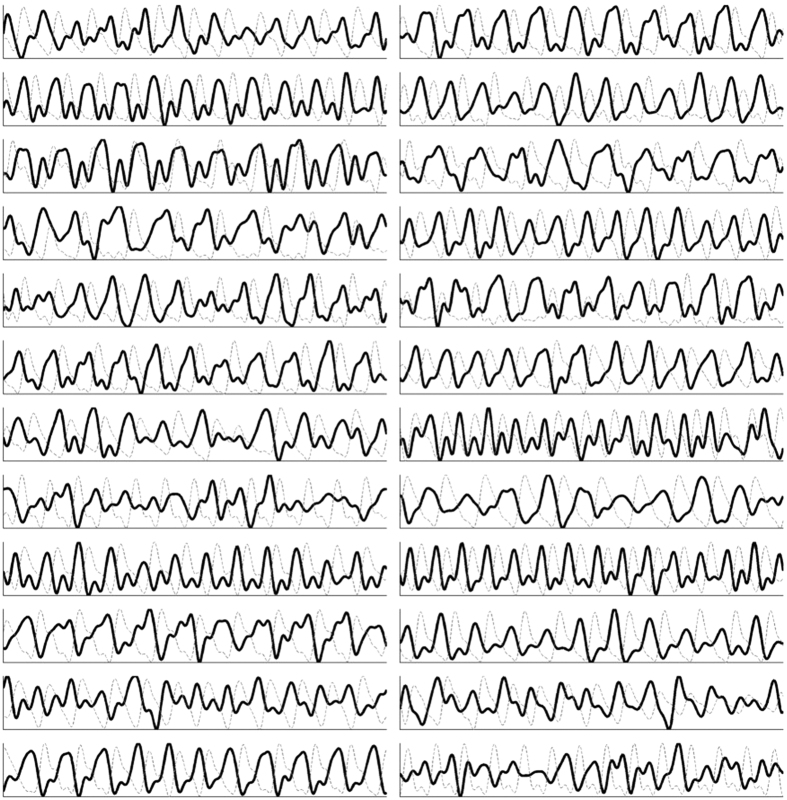
Jugular venous pulse signal acquired for each participant (n = 24). The jugular venous pulse (black line) acquired using the non-contact coded hemodynamic imaging system is compared to the ground-truth arterial waveform (dotted gray). The two waveforms are strongly inversely proportional, some more strongly than others due to factors such as fat content and age-related skin inelasticity.

Characteristics commonly found in major arterial waveforms were observed in the positively correlated waveforms, including a sharp increase in absorbance toward a systolic peak, followed by a dicrotic notch, then a diastolic minimum. The “inverted” waveforms exhibited different characteristics, specifically a gradual rise, followed by a steep drop in absorbance, and often a small fluctuation following the drop. These arterial rises and inverted drops were temporally in sync. Figure 2 shows a typical example of the two types of pulses observed in a participant. The two pulses were strongly correlated through an inverse relationship (*r* = 0.96), and each exhibited strong spatially cohesive patterns. Figure 3 shows the inverted pulse signal acquired for all participants. The hypothesis was that the system was detecting both arterial and venous blood pulses. The following subsections tested this hypothesis.

### Primary anatomical locations of strong pulsing

**Figure 4:**
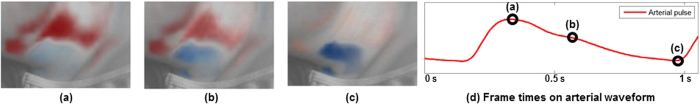
Three frames from a typical segment showing the timing of the blood volume pulse from Supplementary Video 1. (**a**) Upon ventricular ejection, the pulse travels through the carotid arterial track (red), reaching systole; there is no inverted pulsatile flow. (**b**) During the arterial descent towards diastole, the pulse continues to travel through the carotid track, and the start of the inverted pulse can be observed (blue). (**c**) No carotid pulse is observed during diastole, and the inverted pulse experiences its maximum absorbance. Best viewed in colour.

Figure 4 shows selected frames from a video of the flow profiles, Supplementary Video 1. Each frame in the original video capture was overlaid with a colour map indicating pulsatile flow. Signals that exhibited strong positive correlation (see (4)) were coded in red, and those with negative correlation were coded in blue. The opacity strength was computed as *r*^2^(*z, a*_*i*_), where *r* is the correlation. The overlay was processed with a Gaussian smoothing kernel for visualization purposes.

This slow motion video was saved at 1/6 the original frame rate to visually emphasize the pulsatile motion. The two types of flow were easily identified visually due to the phase offset nature of their peaks. Specifically, the arterial track filled with blood simultaneously as the ground truth arterial waveform reached systole. When the arterial waveform reached diastole, the inverted pulse reached its peak. The two tracks exhibited consistent alternating pulsing patterns.

**Figure 5:**
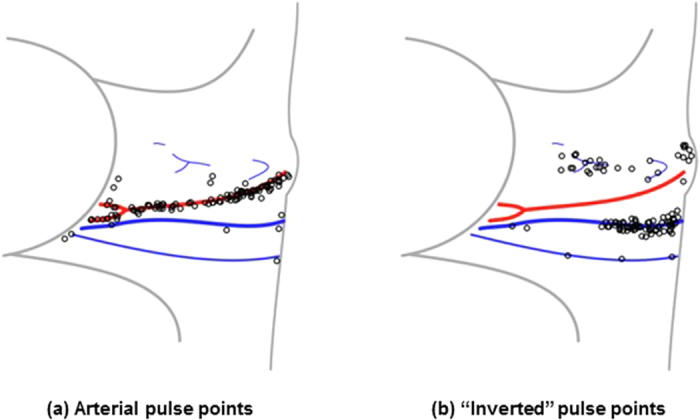
Locations exhibiting the strongest pulsing across all participants (*n* = 24) relative to the position of the carotid artery and jugular vein. Locations were determined by finding and marking the carotid and jugular track using ultrasound following video data capture. The pulsing locations were normalized and drawn relative to the individual’s marked anatomy. The data are presented for the right side only (marked with ‘o’), which is used clinically as the most direct conduit to the heart. The arterial pulse points were consistent with the anatomical location of the carotid artery (**a**), and the inverted waveform pulse points were consistent with the distal location of the jugular vein (**b**). Best viewed in colour.

Figure 5 shows the locations on the neck where the five strongest positive and inverted pulsations occurred for each participant. Following data collection, the carotid and jugular tracks were marked with the guidance of ultrasound. Comparing the marked locations with the major pulsing locations, the arterial pulse locations followed the carotid track. Inverted pulsatile flow was consistently situated on the distal side of the carotid artery. This was consistent with cross-sectional ultrasound imaging, which located the jugular vein on the distal side of the common carotid artery in all participants.

During data analysis, Doppler ultrasound confirmed that the jugular vein was pulsatile in all 24 participants, and cross-sectional ultrasound analysis visually confirmed the phase offset nature of the carotid and jugular pulsing.

### Correlation to jugular venous pulse waveform

**Figure 6:**
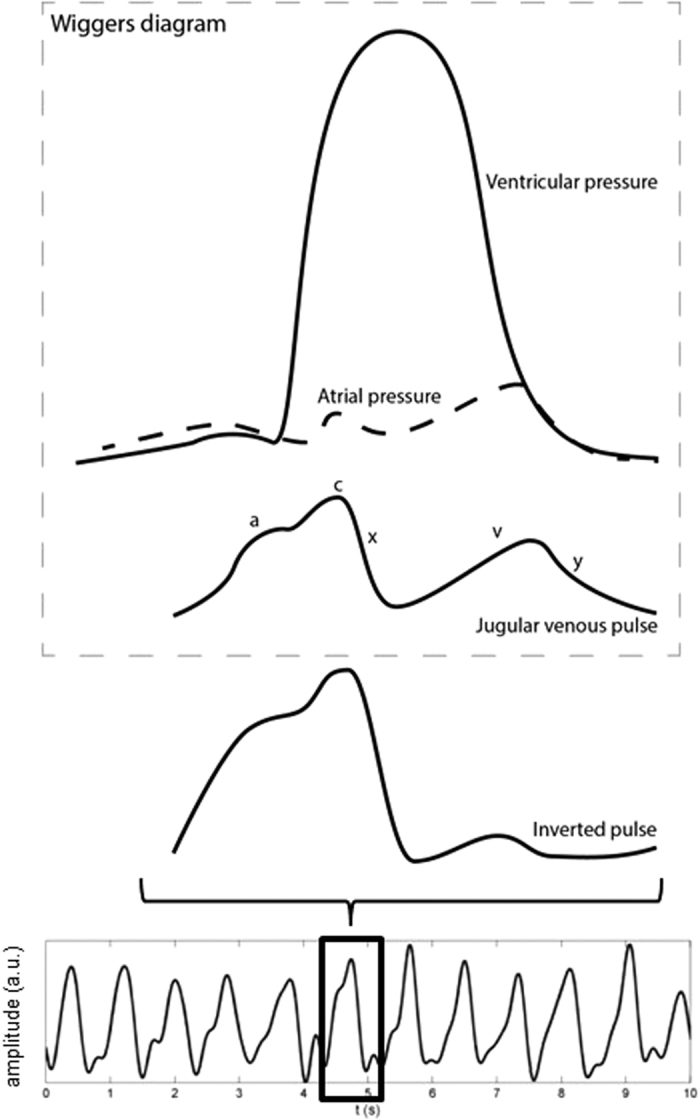
Comparison of a typical “inverted” pulse to the jugular venous pulse waveform in the Wiggers diagram (adapted from ref. 24). The inverted pulse was consistent with the JVP waveform. The JVP waveform is biphasic whose waveform inflections are governed by differential cardiac pressures (see text). The variability between individual pulses is likely due to the effect of respiration on the intrathoracic pressure.

**Figure 7:**
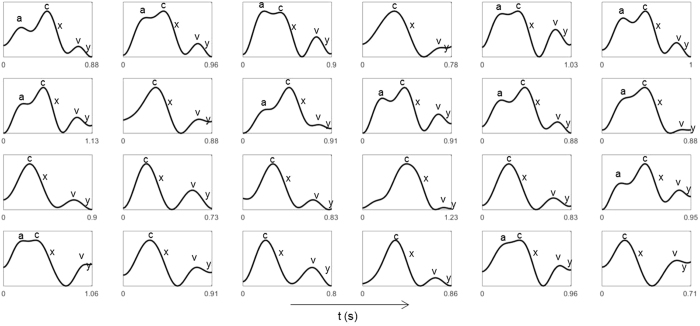
Example of a single pulse extracted from each participant. The characteristic JVP waves were manually identified in each waveform and annotated for clarity. Four of the major JVP waves (c, x, v, y waves) were observed in all participants. The subtle pre-systolic a wave was observed in 13/24 participants.

The inverted blood pulse waveform shape was consistent with the jugular venous pulse (JVP) waveform^19^. Figure 6 shows the time-aligned Wiggers diagram section with the labeled JVP and a typical inverted pulse observed during trials. Visually, the JVP and inverted pulse waveforms show strong intercorrelation. The JVP waveform is biphasic and is characterised by: an increase in pressure pre-systole due to right atrial contraction (*a*); an increase in pressure due to ventricle contraction (*c*); a decrease in pressure during systole due to atrial relaxation following tricuspid valve closure (*x*); an increase in pressure in late systole due to right atrial filling from venous return (*v*); and a decrease in pressure during diastole due to right ventricular filling from the opening of the tricuspid valve (*y*). The inverted pulsing results were consistent with the JVP waveform: gradual increase in blood volume pre-systole (*a*); an increase in pressure during ventricular systole (*c*); a sharp decrease in blood volume was observed slightly prior to the carotid upstroke (*x*); and a small transient rise in blood volume during late systole (*v, y*). Since the JVP is governed by differential pressures generated by heart mechanics, observing the venous pulsation patterns can provide insight into not only vascular function, but aspects of heart function as well, without catheterisation. Figure 7 shows a single pulse waveform for each participant. The *c, x, v* and *y* waves were consistently observed across all participants, and the subtle pre-systolic *a* wave was observed in 13 participants.

## Discussion

These trials support the hypothesis that the PPGI system was able to observe the spatial trajectory of both major arterial and venous blood pulse waveforms in the neck. The amplitude changes of the two signals exhibited different differential properties. In particular, the arterial signal was characterised by a sharp rise during systole followed by a fall during late systole and diastole, whereas the venous signal was characterised by a gradual rise during atrial filling followed by a sharp drop during atrial and ventricular contraction. These findings are consistent with expected transient local blood volume absorption fluctuations^10^, supporting the hypothesis that the data is indeed revealing light-tissue mechanisms rather than a ballistocardiographic mechanisms.

**Figure 8:**
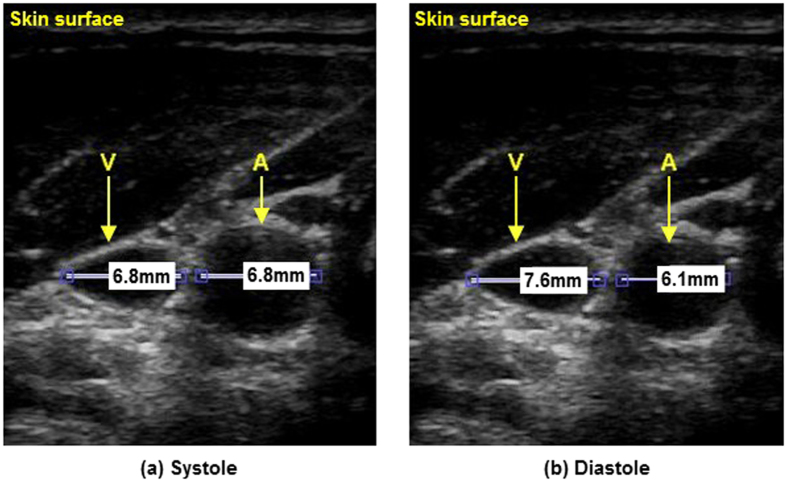
Inverse relationship between the carotid artery and jugular vein as seen with B-mode ultrasound in a typical participant. The jugular vein (below V) and carotid artery (below A) are located below the skin surface (top) with vessel diameters marked in millimetres. Between systole (**a**) and diastole (**b**), the carotid relaxes (10% diameter reduction), and the jugular expands (12% diameter expansion).

The strong negative correlation between the arterial and venous blood pulse waveforms can be attributed to the differential pressure profiles resulting from normal cardiac cycles. The observed results are consistent with the Wiggers diagram. In particular, cardiac contraction ejects blood through the arterial track, and ends with aortic closure. During arterial systole, atrial relaxation causes a decrease in cardiac pressure, resulting in increased venous return into the right atrium, which reduces the volume in the neck veins. Atrial filling pressure gradually increases simultaneously with late systole, resulting in decreased venous return (increased jugular venous volume). This inverted amplitude and phase-offset vessel wall motion was visually confirmed using B-mode ultrasound, where cross-sectional videos showed phase-offset vessel wall expansion and contraction between the carotid artery and jugular vein (see Figure 8). Doppler ultrasound was also used to confirm the pulsatile nature of the jugular vein in the participants consistent with the JVP waveform.

Due to the close proximity of the major neck vessels to the heart, the venous blood pulse waveform can be used to assess heart function. Leveraging the strong correlation between the venous blood pulse waveform and JVP, PPGI can be used to assess heart function that is reflected in the venous waveform in a non-contact manner. Since the jugular vein is a major venous extension of the right atrium, abnormalities in the waveform can indicate heart function problems. For example, the increased pressure from atrial contraction due to tricuspid stenosis produces a larger *a* wave^3^,^20^; a lack of atrial contraction/relaxation from atrial fibrillation inhibits the *a* wave and *x* wave^3^; and blood flow back through the atrium due to tricuspid regurgitation results in a fused *c*-*v* wave^3^. The *c, x, v* and *y* waves were consistently observed in all participants, however the *a* wave was only observed in a subset of participants (13/24). The pre-systolic *a* wave is a subtle signal exhibited by the backflow of venous blood from right atrial contraction. The imaging sensor properties (bit depth, pixel noise magnitude, and frame rate) may have contributed to an undetected *a* wave in individuals with lower central venous blood volume or weaker right atrial contraction. Future work must systematically evaluate the effect of imaging sensor properties on JVP extraction to elucidate this relationship.

Due to a gap in current clinical monitoring technology, potential clinical information encoded in the spatial flow profile is largely unknown. For example, flow velocity and jugular column height can be assessed using the proposed hemodynamic imaging system, which may be important indicators of heart function and central venous pressure^6^,^7^. Clinical studies leveraging the presented work may elucidate the effect of spatial flow profile analysis on heart failure diagnosis and prevention. Future work will investigate the relationship between pulsatile signal characteristics and demographic groups.

This study is the first PPGI study to demonstrate the ability to observe the JVP. Currently, the system is limited to at-rest monitoring with minimal motion, but image processing functionality can be incorporated in future work, including motion compensation sensing^13^. Building on these findings, future work must focus on clinical validation against catheter or ultrasound JVP. A rigorous comparison against current JVP monitoring technology is essential for clinical viability of such a technology to help doctors monitor cardiac dysfunction for rapid visual patient assessment in non-surgical settings. These early findings show promise for shifting from catheter insertion techniques for measuring the JVP waveform toward a non-contact, non-invasive biophotonic imaging solution.

## Methods

### Study protocol

**Figure 9:**
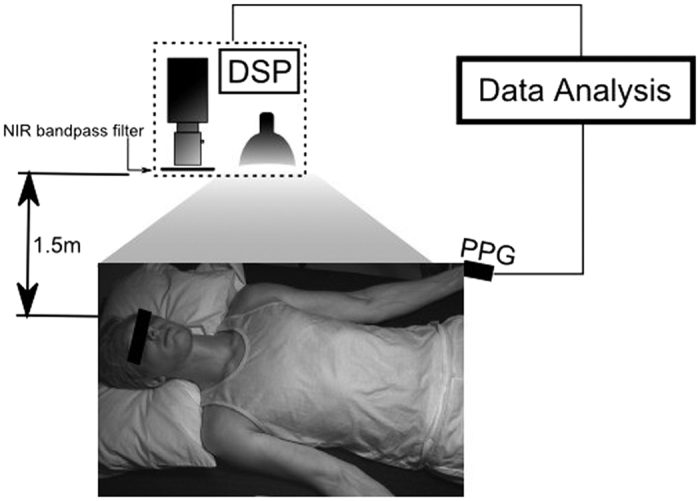
Study setup. Participants were supine for the duration of the study. The biophotonic imaging system was positioned above and slightly to the right of the participant, at a distance of 1.5 m. Illumination was provided by a spatially uniform 250 W tungsten-halogen illumination source. Imaging data were processed on a digital signal processing (DSP) unit. The participant wore a finger cuff which provided the ground truth arterial waveform for the analysis.

Data were collected across 24 participants (age (*μ* ± *σ*) = 28.7 ± 12.4). Figure 9 graphically shows the setup of the study. Demographic information (age, height, weight, body fat %) was obtained at the beginning of the study. The participants were asked to assume a supine position for the duration of the study. To avoid visual occlusion of the neck, the ground truth blood pulse waveform was collected using a finger photoplethysmography (PPG) cuff simultaneously with the video data. The Supplementary Information includes the waveform dataset collected and used in this paper. An ultrasound technician with 14 years’ experience in research ultrasound placed an 11 MHz ultrasound probe (Vivid i, General Electric Healthcare, Horten, Norway) on the neck after video imaging. Upon locating the vessel anatomy, the pressure exerted on the probe was released until the probe broke contact with the skin, and then reapplied gently to the gel interface in the same location to allow undisturbed vessel diameter analysis. Ultrasound videos were collected at 12 fps. B-mode cross-sectional videos were acquired to confirm the location of the jugular vein relative to the carotid artery, and confirm vessel pulsatility characteristics frame-by-frame. The jugular vein was identified by pressing the probe into the skin and observing which of the two vessels collapsed through the cross-sectional view. Longitudinal Doppler measurements were acquired of the jugular vein to validate pulsatile jugular flow. The carotid and jugular paths were marked after video collection to anatomically map the observed pulse’s location. Informed consent was obtained from all participants, and by those participants whose photos were used in this paper. The study was approved by a University of Waterloo Research Ethics committee and performed in accordance with the Declaration of Helsinki.

### Imaging system

A custom photoplethysmographic imaging (PPGI) system was used to collect the data, consisting of a near-infrared sensitive camera (PointGrey GS3-U3-41C6NIR-C), an 850–1000 nm optical bandpass filter, and a 250 W tungsten-halogen illumination source. The spectral bandwidth was selected within the tissue optical window, which exhibits deep photon penetration (to reach major vessels) and low melanin absorption (skin tone insensitivity)^21^. The illumination was secured at a fixed distance using a light stand to ensure stable skin irradiance, and it was uniformly projected using a 16” glass fabric front diffuser. Both the illumination source and imaging system were situated 1.5 m above the participant. Pixel distances were pre-calibrated using a resolution target at the known fixed imaging distance. Videos were collected at 60 fps, using 16 ms exposure time and f4.0 aperture. The data were processed using a digital signal processing (DSP) unit. In one case, the participant’s neck was not visible from overhead, so a bedside view was used.

**Figure 10:**
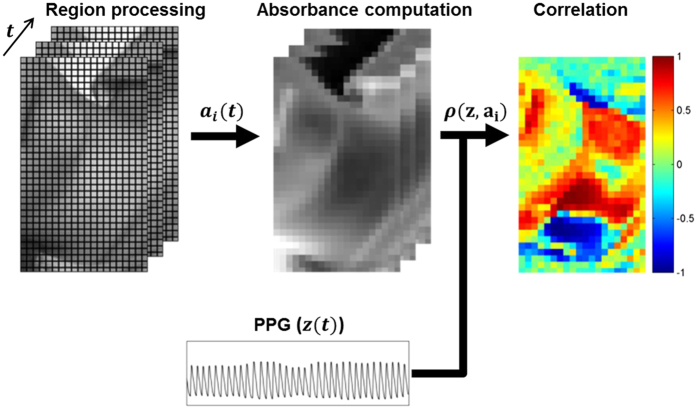
Data processing pipeline. Each frame was analysed in 0.25 × 0.25 mm regions. Each region’s temporal fluctuations were converted to absorbance using (2). Pearson’s linear correlation coefficient was computed for each waveform using the ground truth PPG waveform, yielding a spatial correlation map showing locations exhibiting strong forward and inverted pulsing. Best viewed in colour.

Figure 10 shows the signal processing pipeline for the study. Each frame was blockwise averaged using 5 × 5 mm regions. The temporal fluctuations of region *i* yielded a reflected illumination signal *x*_*i*_(*t*):


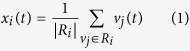


where *R*_*i*_ is the 5 × 5 mm pixel region surrounding pixel *i*, and *v*_*j*_(*t*) is the *j*^th^ pixel value at time *t*. Reflectance was converted to absorbance using Beer-Lambert law, modeling the geometrical photon path as the scattered reflected path^22^:


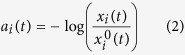


where 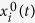 denotes the theoretical illumination incident on the tissue at time *t*. A detrending method^23^ was used to eliminate environmental illumination variations. Thus, 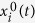 was considered to be temporally stable (i.e., 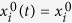), and the unitless nature of the blood pulse waveform resulted in a final formulation of *a*_*i*_(*t*):


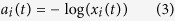


### Data analysis

Using the finger PPG signal as the ground truth arterial blood pulse waveform, Pearson’s linear correlation coefficient was computed between each region’s temporal absorbance signal *a*_*i*_(*t*) and the PPG signal *z(t*) to determine the signal strength and directionality:


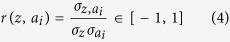


where *σ*_*z*_, 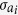 are the standard deviation of the PPG sensor and region signals respectively, and 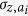 is the covariance of the two signals. Note that this is temporal correlation, not scatter data correlation, where *r* > 0.5 indicates strong pulsing consistent with the PPG arterial pulsing signal, and *r* < −0.5 indicates strong pulsing that is inversely proportional to the PPG signal. For visualisation purposes, the signals were filtered using an ideal bandpass filter from 0.5–3.5 Hz (30–210 bpm), and the colour maps were smoothed using a Gaussian kernel (*σ* = 2.5 mm).

In some cases (e.g., cold fingers), the carotid arterial waveform’s shape differed substantially from the finger PPG waveform. In these cases, the waveform that exhibited the strongest spectral signal-to-noise ratio (SNR) with respect to the PPG waveform was used instead. SNR was calculated in the frequency domain across all regions as:


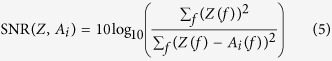


where *Z, A*_*i*_ are the zero-DC normalized spectral magnitudes of the PPG and *i*^th^ region signals, respectively, and *f* represents frequency. The template waveform was then chosen as follows:


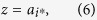



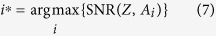


## Additional Information

**How to cite this article:** Amelard, R. *et al*. Non-contact hemodynamic imaging reveals the jugular venous pulse waveform. *Sci. Rep.* **7**, 40150; doi: 10.1038/srep40150 (2017).

**Publisher's note:** Springer Nature remains neutral with regard to jurisdictional claims in published maps and institutional affiliations.

## Supplementary Material

Supplementary Information

Supplementary Video 1

Supplementary Dataset 1

## Figures and Tables

**Table 1:** Summary of sample demographics.

Demographic	Sample Representation
n (female/male)	24 (11/13)
age (years)	9–60 (28.7 ± 12.4)*
body fat (%)	10.5–42.3 (21.0 ± 7.9)*
muscle (%)	31.0–53.9 (40.4 ± 5.3)*
BMI (kg · m^−2^)	16.4–35.1 (25.5 ± 5.2)*

^*^μ ± *σ*.
