# Supplementary material for: Non-contact hemodynamic imaging reveals the jugular venous pulse waveform
Source: Sci Rep. 2017 Jan 9;7:40150. doi: 10.1038/srep40150 (PMC5220303; doi:10.1038/srep40150)
Supplement: Supplementary Information [file srep40150-s1.pdf]

Supplementary Information for

Non-contact hemodynamic imaging reveals the jugular venous pulse waveform

Robert Amelard, Richard L Hughson, Danielle K Greaves, Kaylen J Pfisterer, Jason Leung, David A Clausi, Alexander Wong

Supplementary Video 1: Slow motion video showing the detected arterial (red) and venous (blue) pulsations through the neck in a typical participant. The two pulses occur alternately out of phase, consistent with ultrasound findings. The relative locations of the two pulses were consistent with the ultrasound findings of the carotid artery and jugular vein.

Supplementary Dataset 1: Dataset used to generate the results of the paper. The waveforms for each participant that were used in the results of the study are provided.
